# Supplementary material for: Detecting early signs of the 2007–2008 crisis in the world trade
Source: Sci Rep. 2016 Jul 27;6:30286. doi: 10.1038/srep30286 (PMC4962096; doi:10.1038/srep30286)
Supplement: Supplementary Information [file srep30286-s1.pdf]

Detecting early signs of the 2007-2008 crisis in the world trade - Supplementary Information

Fabio Saracco[1,3], Riccardo Di Clemente[2*,3], Andrea Gabrielli[1,3], and Tiziano Squartini[1,3]

[1]IMT Institute for Advanced Studies, Lucca, 55100, Italy
[2]Massachusetts Institute of Technology, Department of Civil and Environmental Engineering, Cambridge, 02139, MA USA
[3]Institute for Complex Systems (ISC-CNR) UOS Sapienza, "Sapienza" University of Rome, Rome, 00185, Italy
[*]rdicle@mit.edu

ABSTRACT

Supplementary Information for the paper "Detecting early signs of the 2007-2008 crisis in the world trade".

The Revealed Comparative Advantage (RCA) threshold

In order to retain only the relevant export data, we adopted the Revealed Comparative Advantage (RCA) threshold[1]

$$\mathrm{RCA}_{cp} \equiv \frac{\frac{q_{cp}}{\sum_{p'} q_{cp'}}}{\frac{\sum_{c'} q_{c'p}}{\sum_{c'p'} q_{c'p'}}} = \frac{q_{cp}}{d_c^w}\frac{W}{u_p^w}, \tag{S1}$$

where q_{cp} is the amout of product p exported by country c and the sums $\sum_{p'} q_{cp'} = d_c^w$ and $\sum_{c'} q_{c'p} = u_p^w$ indicate, respectively, the weighted diversification of country c[1] and the weighted ubiquity[1] of product p. The recipe provided by RCA allows us to select the product p whose impact on the export basket of country c is larger than the impact of p on the global market. If $\mathrm{RCA}_{cp} \geq 1$, $q_{cp}/d_c^w \geq u_p^w/W$ and the corresponding entry of the binary matrix $\mathbf{M}$ is $m_{cp} = 1$; on the other hand, $\mathrm{RCA}_{cp} < 1$ implies that $m_{cp} = 0$.

Additional sectors analysis

Although the macrosectors "manufacture of chemicals", "manufacture of fabricated metal products" and "textile" are characterized by the highest values of internal similarity (thus matching better the definition of "macrosectors" in the economic jargon), they seem to be insensitive to the approaching crisis, showing remarkably stable tendencies throughout our whole temporal window (see fig. S1). As shown in the section Results, the most informative sectors on the approaching crisis are those characterized by a moderately high level of internal similarity.

Additional measures for binary, undirected, bipartite networks

In the following subsection, we provide the explicit definition of two additional topological quantities, in order to corroborate our findings with the analysis of more traditional network quantities. By applying the same kind of analysis presented in the main text, we show that early-warning signals of the crisis are detectable even analysing nestedness and assortativity.

Nestedness. Loosely speaking, the degree of "triangularity" of the observed biadjacency matrix can be quantified according to a number of measures recently proposed under the common name of nestedness.[3–6] Here we adopt the one proposed in,[3] called NODF, an acronym for "Nestedness metric based on Overlap and Decreasing Fill".

Since the total value of nestedness (NODF_t) is the weighted average of the contribution from rows (NODF_r) and the contribution from columns (NODF_c), we have considered all of them for the present analysis. Naturally, the adopted measure of nestedness does not depend on the rows and columns ordering criterion.[3] If we indicate with N_c the number of columns and with N_r the number of rows, then

$$\mathrm{NODF}_t \equiv \frac{N_c(N_c-1)\mathrm{NODF}_c + N_r(N_r-1)\mathrm{NODF}_r}{N_c(N_c-1) + N_r(N_r-1)} \tag{S2}$$

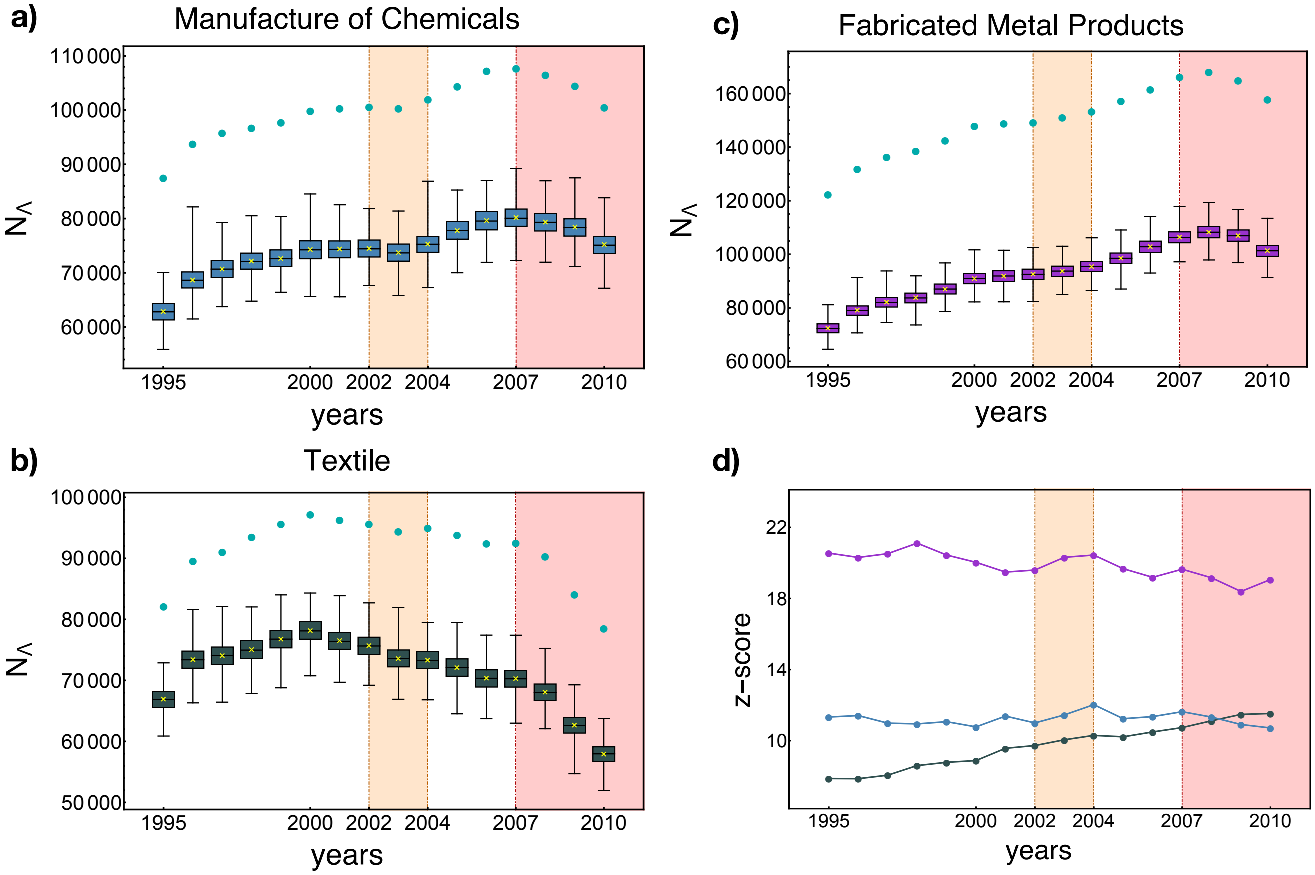

Figure S1. Evolution of restricted Λ-motis across the period 1995-2010. Panels a), b) and c) show the box plots of the Λ-motifs of sectors • - manufacture of chemicals, • - manufacture of fabricated metal products and • - textile across the considered time window. The mean of the year-specific BiCM-induced ensemble distribution is indicated as a yellow cross; light-blue dots represent the observed abundances of motifs. Panel d) shows the evolution of the motifs z-scores (lines joining the dots simply provide a visual aid). The macrosectors characterized by the highest values of internal similarity seem to be insensitive to the approaching crisis, showing remarkably stable (increasing or decreasing) tendencies throughout our whole temporal window.

whose row- and column-specific contributions are provided by

$$\mathrm{NODF}_c \equiv \frac{2\sum_{c<c'} T^C_{cc'}}{N_c(N_c-1)};\ \mathrm{NODF}_p \equiv \frac{2\sum_{p<p'} T^P_{pp'}}{N_p(N_p-1)}, \tag{S3}$$

where $T^C_{cc'} = \frac{\sum_p m_{cp} m_{c'p}}{\min[d_c, d_{c'}]}$ (if $d_c \neq d'_c$ and 0 otherwise) and $T^P_{pp'} = \frac{\sum_c m_{cp} m_{cp'}}{\min[u_p, u_{p'}]}$ (if $u_p \neq u'_p$ and 0 otherwise).[3]

Assortativity coefficient. The second global quantity we have considered to characterize the WTW evolution is the assortativity measure $r \in [-1,1]$ proposed in,[7] with $r = 1$ indicating the maximum observable correlation between degrees (thus measuring the strongest tendency of links to connect nodes with similar degrees) and $r = -1$ indicating the minimum observable correlation between degrees (thus measuring the strongest tendency of links to connect nodes with different degrees).

The definition of the assortativity coefficient can be found in.[7] By making it explicit which links contribute to the sums at the numerator and at the denominator, such a coefficient can be rewritten more clearly in terms of the bipartite nodes degrees, as

$$r \equiv \frac{4L\left(\sum_{c,p} m_{cp} d_c u_p\right) - \left(\sum_c d_c^2 + \sum_p u_p^2\right)^2}{2L\left(\sum_c d_c^3 + \sum_p u_p^3\right) - \left(\sum_c d_c^2 + \sum_p u_p^2\right)^2}. \tag{S4}$$

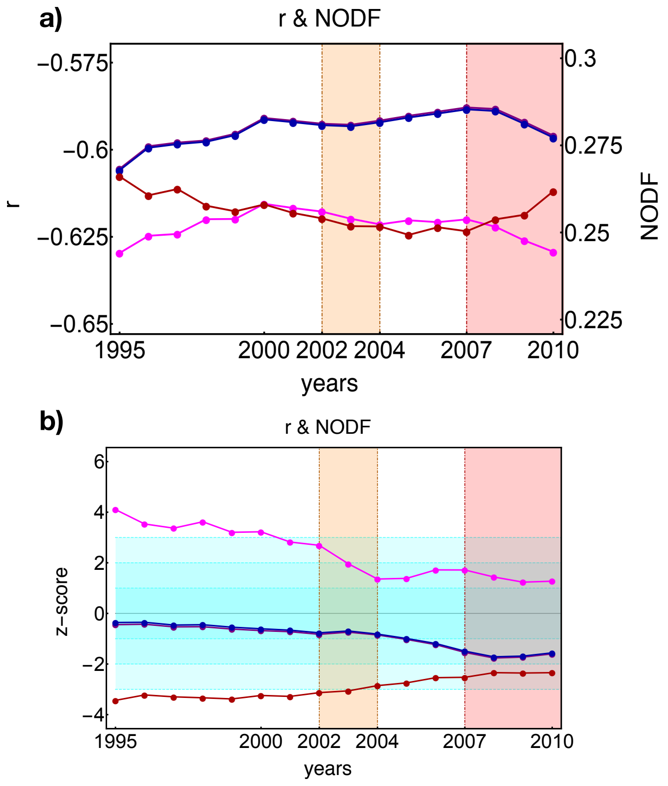

Figure S2. Evolution across the period 1995-2010 of the total nestedness - •, the row-specific nestedness - •, the column-specific nestedness - • and the assortativity - •. Panel a) shows the evolution of the observed abundances; panel b) shows the evolution of the corresponding z-scores, pointing out the peculiarity of the year 2003 in discriminating between a statistically significant phase (1995-2003) and a phase consistent with our null model (2003-2007). Lines joining the dots simply provide a visual aid

.

Nestedness and Assortativity analysis. Fig. S2a shows the evolution of the observed abundances of nestedness ($NODF_t$ in blue; $NODF_r$ in pink; $NODF_c$ in purple) and assortativity (in brown). In particular, all of them provide evidence of the peculiar character of year 2007: all trends, in fact, even if characterized by opposite behaviors as the row-specific NODF and assortativity, show an inversion in correspondence of it.

Let us now carry on the comparison between the aforementioned observed trends and their expected counterparts under the BiCM, by plotting the corresponding z-scores. As shown by fig. S2b, $NODF_t$ and $NODF_c$ are always consistent with our null model, even if they gradually come closer to the $z = -2$ border as 2007 approaches. Again, the rate of increase of randomness across and after 2003 is notable. In particular, the total NODF spans one sigma of statistical significance in just four years (2003-2007), the same range having been spanned across the previous nine years.

As for their observed counterparts, the assortativity z-score is characterized by an opposite trend; in particular, it provides a clear statistical signal in 2003 by crossing the significance bound $z = -3$. This means that the degree of assortativity of the network becomes less and less significant, to become compatible with the BiCM-induced random value four years before 2007; it then steadily rises from 2003 until 2008 and seems to maintain such a value afterwards.

A more evident signal, confirming the increasingly random character of the network, is provided by the z-score of the row-specific NODF, whose analysis allows us to clearly distinguish two distinct phases, the first one lasting from 1995 to 2003 (characterized by a decreasing trend) and the second one lasting from 2003 to 2010 (characterized, instead, by an almost constant trend). The biennium across 2003 seems to constitute a somehow crucial period, defined by a decrease of statistical significance of two sigmas (from $z \simeq 3$ to $z \simeq 1$); analogously to what already observed for $NODF_t$, the same loss of statistical significance (from $z \simeq 4$ to $z \simeq 2$) was spanned by $NODF_r$ in the preceding eight years.

As for motifs, the z-scores trends considered so far agree in pointing out the peculiarity of 2003 as the year in which the network gets through two different regimes, from a "structured" phase, not compatible with our null model, to a increasingly

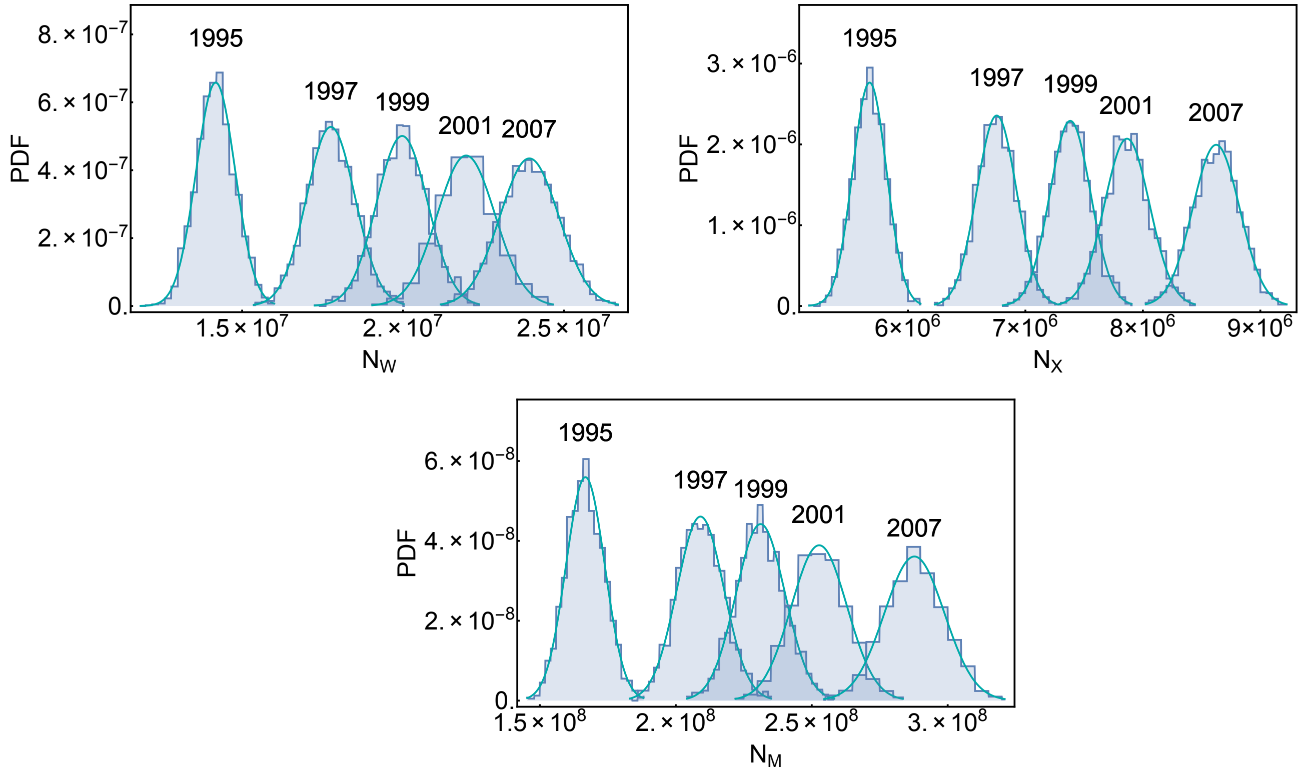

Figure S3. Comparison of the ensemble distributions of our X-, W- and M-motifs with normal distributions having the same mean and variance, for different years of our data set. The very good agreement obtained justifies the computation of the z-score for the abundances of such motifs.

random phase, where nodes correlations become increasingly similar to their random counterpart.

References

1. Tacchella A., Cristelli M., Caldarelli G., Gabrielli A. & Pietronero L. A New Metrics for Countries' Fitness and Products' Complexity. *Sci. Rep.* **2**(723), doi:10.1038/srep00723 (2012).
2. http://www.unstats.un.org/unsd/cr/registry/regcst.asp?Cl=8.
3. Almeida-Neto M., Guimaraes P., Guimaraes P. R. Jr., Loyola R. D. & Ulrich W. A consistent metric for nestedness analysis in ecological systems: reconciling concept and measurement. *Oikos* **8**(117), 1227-1239 (2008).
4. Bastolla U., Fortuna M. A., Pascual-Garcia A., Ferrera A., Luque B. & Bascompte J. The architecture of mutualistic networks minimizes competition and increases biodiversity. *Nature* **7241**(458), 1018-1020 (2009).
5. Staniczenko P. P. A., Kopp J. & Allesina S. The ghost of nestedness in ecological networks. *Nat. Comm.* **4**(139), doi:10.1038/ncomms2422 (2013).
6. Jonhson S., Dominguez-Garcia V. & Munoz M. A. Factors Determining Nestedness in Complex Networks. *PLoS ONE* **8**(9), doi:10.1371/journal.pone.0074025 (2013).
7. Newman M. E. J. Assortative mixing in networks. *Phys. Rev. Lett.* **89**, 208701 (2002).

Acknowledgements

This work was supported by the EU project GROWTHCOM (611272), the Italian PNR project CRISIS-Lab and the EU project SIMPOL (grant num. 610704). The authors acknowledge Andrea Tacchella for the data cleaning and Giulio Cimini, Matthieu Cristelli, Emanuele Pugliese and Antonio Scala for useful discussions.

Author contributions statement

FS and RDC analysed the data and prepared all figures. AG wrote the article. TS planned the research and wrote the article. All authors reviewed the manuscript.

Additional information

The Authors declare non competing financial interest. The funders are no role in study design, data collection and analysis, and decision to publish the manuscript.
